# Elucidating unconscious processing with instrumental hypnosis

**DOI:** 10.3389/fpsyg.2014.00785

**Published:** 2014-07-28

**Authors:** Mathieu Landry, Krystèle Appourchaux, Amir Raz

**Affiliations:** ^1^Integrated Program in Neuroscience, McGill UniversityMontreal, QC, Canada; ^2^Department of Psychiatry, McGill UniversityMontreal, QC, Canada; ^3^Lady Davis Institute for Medical Research, Jewish General HospitalMontreal, QC, Canada

**Keywords:** unconscious, instrumental hypnosis, suggestion, subliminal perception, preconscious processing, suppression of consciousness, consciousness, global workspace

## Abstract

Most researchers leverage bottom-up suppression to unlock the underlying mechanisms of unconscious processing. However, a top-down approach – for example via hypnotic suggestion – paves the road to experimental innovation and complementary data that afford new scientific insights concerning attention and the unconscious. Drawing from a reliable taxonomy that differentiates subliminal and preconscious processing, we outline how an experimental trajectory that champions top-down suppression techniques, such as those practiced in hypnosis, is uniquely poised to further contextualize and refine our scientific understanding of unconscious processing. Examining subliminal and preconscious methods, we demonstrate how instrumental hypnosis provides a reliable adjunct that supplements contemporary approaches. Specifically, we provide an integrative synthesis of the advantages and shortcomings that accompany a top-down approach to probe the unconscious mind. Our account provides a larger framework for complementing the results from core studies involving prevailing subliminal and preconscious techniques.

## INTRODUCTION

The unconscious mind fascinates and challenges human thinking (Tallis, 2002). Pervasive even in popular science (Mlodinow, 2012), the so-called “new” unconscious shares in the innovations and advances of consciousness research (Dehaene et al., 2006; Kouider and Dehaene, 2007; Seth et al., 2008; Dehaene, 2011; Dehaene and Changeux, 2011). This fast-growing field offers novel perspectives concerning the powerful influence of the unconscious mind on thought and behavior (Hassin et al., 2005). In the quest to understand the unconscious realm, various psychophysical techniques that suppress conscious access to sensory events largely frame our insights regarding the depth of unconscious processing and serve as a robust methodological backbone (Kim and Blake, 2005). Yet, despite such valuable methods, inconsistencies across tasks fuel a conundrum regarding the depth of processing of the cognitive unconscious – unconscious mental structures and processes that support thoughts and behaviors (Kihlstrom, 1987). These inconsistencies not only call for caution when generalizing results from a single family of similar tasks, but also suggest that suppression mechanisms are mostly task-dependent (Tsuchiya et al., 2006; Faivre et al., 2014; Fogelson et al., 2014; Izatt et al., 2014). In their attempt to identify the underlying mechanisms subserving unconscious processing, researchers increasingly seek to diversify their critical inquiry. Here we draw upon the science of hypnosis – a technique with a long track record of study concerning the unconscious – and show how it can become a useful vehicle to complement and diversify existing empirical approaches.

Recovering from a volatile history plagued by quackery and charlatanism, hypnosis has become a viable venue of cognitive science (Oakley and Halligan, 2009, 2013; Raz, 2011b). At least in part, this interest owes to the potent influence hypnotic and post-hypnotic suggestions wield over sensory, cognitive, and motor processing (Nash and Barnier, 2008). Relying on such findings, we argue that research on the cognitive unconscious would benefit from including hypnosis paradigms. Complementing current assortment of suppression techniques with the powerful effects of hypnosis affords researchers with a distinctive mean to test novel hypotheses about unconscious processing.

Using hypnosis in the study of the unconscious mind dates back to early psychodynamic conceptions when analysts leveraged hypnotism to probe unconscious thoughts and feelings of analysands (Bachner-Melman and Lichtenberg, 2001). Revisiting this idea, hypnosis research informs our scientific views of the cognitive unconscious, mental processes, and their structure (Kihlstrom, 1987). Here we draw on this framework and outline how instrumental hypnosis – i.e., the instrumental use of hypnotic suggestions to explore the underlying mechanisms of typical and atypical cognition – promises to make way for a top-down approach in the study of unconscious processes. Specifically, this top-down approach aims to harness the effects of higher cognitive functions upon lower level processing. We argue that instrumental hypnosis paves the road to multiple methodological advances in the exploration of the unconscious mind. We differentiate between subliminal and preconscious approaches (Dehaene et al., 2006; Dehaene, 2011), whereby the former reflects perceptual failures and the latter attentional failures (Kanai et al., 2010). We will explain how hypnotic suggestions can exploit the mechanisms of suppression and inattention to unravel unconscious processes. Importantly, this innovative framework does not champion top-down over bottom-up approaches, but rather advocates exploiting both approaches together to better unravel the complexity of unconscious processing.

We review contemporary suppression and inattention techniques to assess their relative merits and drawbacks. Thereafter, we contrast the strengths and weaknesses of contemporary approaches – i.e., subliminal and preconscious methods – with those of instrumental hypnosis. Showcasing findings using hypnosis, we sketch out how this top-down approach provides the experimental means to foster new perspectives to study the unconscious mind.

## PART I – MODERN CONCEPTIONS OF THE UNCONSCIOUS MIND AND THE GLOBAL WORKSPACE THEORY OF CONSCIOUSNESS

Subliminal and preconscious approaches represent active areas of research within the domain of unconscious cognition (Kim and Blake, 2005; Kouider and Dehaene, 2007; Jensen et al., 2011). Guided by various techniques designed to eliminate conscious access of sensory events (Kim and Blake, 2005), subliminal research gave way to the emergence of different theories (Hassin et al., 2005). Critically, conceptions of the unconscious mind remain largely contingent on current theories of consciousness: engaging unconscious perception entails disrupting at least one mechanism that would otherwise enable conscious perception (Dehaene et al., 2006; Kanai et al., 2010; Dehaene, 2011; Dehaene and Changeux, 2011). In the global workspace theory of consciousness, the progression from unconsciousness to consciousness proceeds from the coordinated interplay between multiple local systems forming an overarching network. More specifically, this model posits that conscious perception stems from the bottom-up propagation of sensory signals across various systems, while top-down processes boost the strength of these signals, enabling global broadcast of information through a virtual forum (Baars, 1988, 2005; Dehaene et al., 1998, 2001, 2003, 2006; Dehaene and Naccache, 2001; Dehaene and Changeux, 2005, 2011; Del Cul et al., 2007; Dehaene, 2011). Therefore, according to this account, consciousness corresponds to a stable state that emerges from the coherent and synchronous activities among distant local processing systems.

**FIGURE 1:**
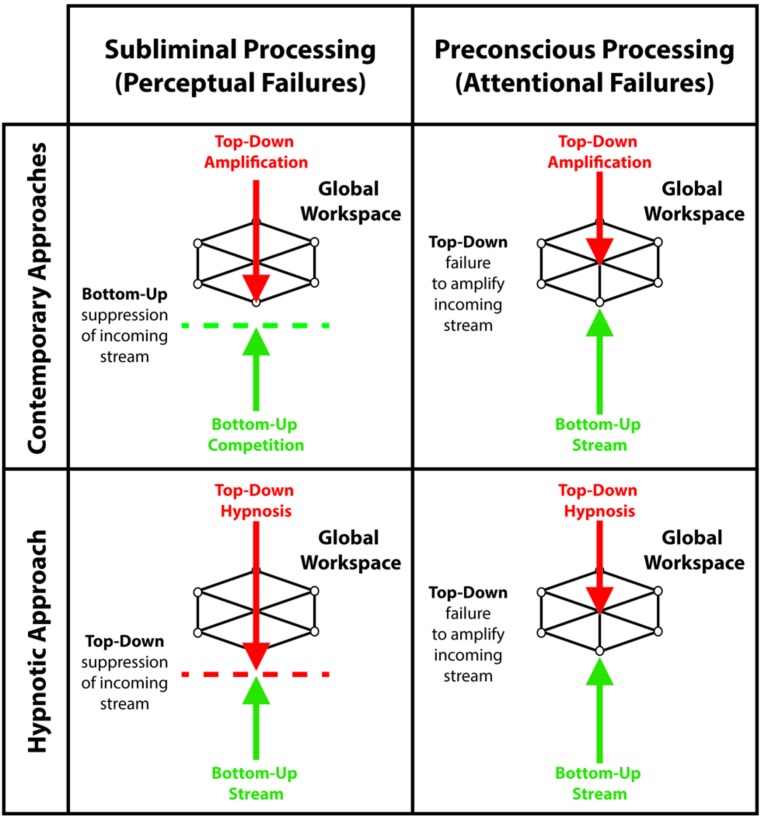
**Contemporary approaches and the hypnotic approach as a function of the taxonomy that differentiates subliminal processing, reflecting perceptual failures, from preconscious processing, reflecting attentional failures.** During *subliminal processing*: contemporary approaches utilize bottom-up competition between sensory inputs to exploit the limits of perception, prevent global broadcast of incoming signals and induce conscious suppression; while the hypnotic approach harness top-down processes to modulate lower perceptual processes and suppress sensory inputs. During *preconscious processing*: both contemporary approaches and the hypnotic approach prevent global broadcast by hindering top-down amplification of incoming sensory signals.

The global workspace model entails that unconscious processing of sensory events occurs in two ways: conscious suppression of sensory signals, corresponding to perceptual failures, and preconscious processing of sensory events reflecting attentional failures (see **Figure 1**; Dehaene et al., 2006; Kanai et al., 2010; Dehaene and Changeux, 2011). During suppression, interruptions of the sensory signal can potentially occur at different stages of sensory processing, leading to subliminal processing. For example, backward masking – a common suppression technique – likely achieves suppression of consciousness by interfering with local boosting processes of sensory signals, which reduces its overall efficiency for global broadcast (Kouider and Dehaene, 2007). During preconscious processing, various techniques divert attention and top-down amplification processes away from sensory events, thereby preventing global broadcast of information and conscious perception. Several experiments report that individuals remain unaware of unattended events (Simons and Levin, 1997; Mack and Rock, 1998; Simons, 2000). Apart from providing significant information about the influences of subliminal and preconscious processing on cognitions and behaviors, both approaches show that understanding the inner workings of the unconscious mind may echo our views on consciousness. Here we unravel the merits and drawbacks of suppression and inattention techniques through the lens of the global workspace model while putting forward the idea that hypnosis may contribute and extend the range of experimental possibilities to study conscious suppression and the unconscious mind.

## PART II – CONTEMPORARY APPROACHES TO THE STUDY OF THE COGNITIVE UNCONSCIOUS

### SUBLIMINAL SUPPRESSION TECHNIQUES – PERCEPTUAL FAILURES

#### Interocular suppression techniques

**FIGURE 2:**
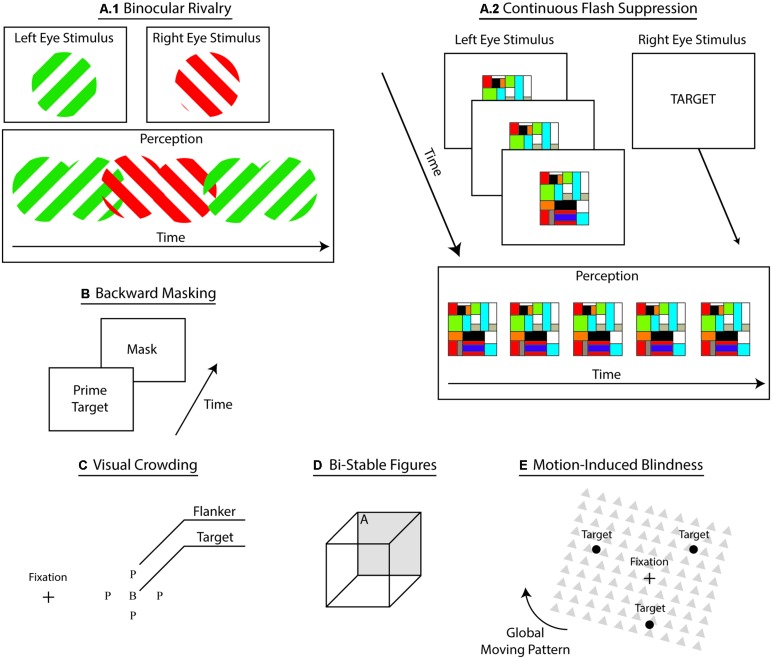
**Subliminal techniques.** A sketch of the prevailing techniques used to suppress conscious perception of sensory inputs. **(A1)** Binocular rivalry where dichoptic presentation of dissimilar stimuli generates fluctuation in conscious perception between representations. **(A2)** Continuous flash suppression where presentation a repeatedly flashed stimulus to one eye induces conscious suppression of static stimulus presented in the other eye.**(B)** Backward masking where rapid sequential presentation of a prime and a mask conscious induces conscious suppression of the prime. **(C)** Visual crowding where flankers interfere with processing of the target in peripheral vision, rendering certain target-related characteristics unrecognizable. **(D)** Bistable figures induce perceptual fluctuations between mutually exclusive visual interpretations – e.g., side A facing upward and then facing downward. **(E)** Motion-induced blindness where movement of the global pattern suppresses conscious perception of the targets.

Interocular suppression refers to an assortment of psychophysical techniques that induce conscious suppression of sensory input through the simultaneous dichoptic presentation of dissimilar stimuli (see **Figure 2**). In this procedure, both stimuli compete to access consciousness, resulting in the temporary conscious suppression of the ineffective stimulus (Blake, 2001; Blake and Logothetis, 2002; Lin and He, 2009; Blake et al., 2014). During binocular rivalry (BR), participants experience transient, yet unpredictable, switches between perceptions of each monocular stimulus. Flash suppression (Wolfe, 1984) and continuous flash suppression (CFS; Tsuchiya and Koch, 2005) techniques aid to overcome this particular shortcoming by governing stimulus onset, thus controlling perceptual dominance and visual awareness. During CFS, experimenters repeatedly flash a single monocular stimulus – i.e., typically high contrast Mondrian patterns – to induce steadier perceptual dominance (See **Figure 2**), which elicits longer and deeper suppression compared to BR (Tsuchiya et al., 2006). Evidence suggests that adaptation represents a central mechanism of perceptual suppression (Kang and Blake, 2010). Some propose that greater suppression during CFS follows from the reduction of neural adaptation (Tsuchiya et al., 2006; Yang and Blake, 2012). However, it remains unclear whether CFS merely represents a form of BR (Tsuchiya et al., 2006; Shimaoka and Kaneko, 2011). Plus, a recent review of BR casts doubts concerning the potential of this technique to provide researchers with critical information about consciousness (Blake et al., 2014). This review underscores concerns related to the validity of control conditions for BR, the distinction between attention and awareness during BR, the generalizability of findings with BR, and the comparison between the neural correlates of BR and the neural correlates of consciousness (NCC). Indeed, according to the authors, instead of indicating the neural mechanisms involved in awareness, multistable techniques – and ensuing transient perceptual changes – could be highlighting perceptual decision processes. In accordance with this criticism, CFS has widely gained in popularity (cf., this *Frontiers in Psychology* research topic on conscious suppression). Importantly, interocular suppression techniques yield competition at the sensory level and at the representational level (Sterzer et al., 2009b), presumably reflecting corresponding changes a the neural level (Sterzer et al., 2014). Accordingly, most accounts explain interocular suppression of consciousness through inhibitory competition at different levels of processing – i.e., lower-level sensory signal and higher-level representations (Tong et al., 2006). This family of techniques provides the critical advantage of inducing suppression under constant visual input, a methodological feature that permits more reliable comparisons of conscious and unconscious perception without confounding variables related to changes in sensory events.

#### Backward masking

A popular suppression approach, visual backward masking eliminates conscious access through rapid sequential presentations of stimuli – a prime target and a mask – that result in the conscious suppression of the prime target (see **Figure 2**; Breitmeyer, 2007; Kouider and Dehaene, 2007). Subliminal processing of masked primes show perceptual, cognitive, and ideomotor effects (Breitmeyer and Ögmen, 2006). A dominant view on backward masking proposes that the mask stimulus suppresses conscious access by interfering with local re-entrant signals that boost sensory signals (Breitmeyer, 2007). Thus, by interrupting this boosting process, masking weakens the sensory signal, rendering it impotent for global broadcast and conscious perception (Dehaene et al., 2006; Dehaene and Changeux, 2011). Critically masking reliably interrupts conscious access to sensory signals, yet suppression remains sensitive to various prime-related and experimental-related factors, such as the type of task, novelty of the prime, category of the prime, etc. (Van den Bussche et al., 2009b). Despite advantageous experimental qualities such as flexibility, generalizability, and robustness, backward masking achieves conscious suppression through the disruption of the visual input – i.e., mask interference. This drawback precludes direct contrast between the conscious and unconscious conditions, which differ in sensory processing, thereby limiting our ability to tease apart the NCC with this approach.

#### Visual crowding

In peripheral vision, nearby distractors – e.g., flankers – render targets unrecognizable (see **Figure 2**; Cavanagh, 2001; Levi, 2008; Whitney and Levi, 2011). This crowding phenomenon aids in uncovering the underlying mechanisms of conscious recognition and object identification (Levi, 2008; Whitney and Levi, 2011). Critically, crowding rarely abolishes conscious access to sensory inputs because target detection remains largely unaffected (Pelli et al., 2004). Instead, crowding capitalizes on the poor resolution of peripheral vision combined with competing noise – e.g., from the flankers – to make the features of the target less discernible (Nandy and Tjan, 2007). The lack of complete suppression of awareness highlights the difficulty in separating subliminal perception from consciousness (Kim and Blake, 2005). Also, similar to backward masking, visual crowding elicits suppression of consciousness through variations of sensory input – i.e., by adding flankers – which further limits our ability to isolate the NCC. Different theories currently compete to explain the effects of visual crowding. According to one such account, the suppression of certain target features proceeds from multilevel interactions comprising a bottleneck situated between lower level features detection and higher order integration processes (Parkes et al., 2001; Levi, 2008; Whitney and Levi, 2011). Supporting this view, the effect of this bottleneck at the integration level shows that targets can systematically acquire certain distractor-related features (Greenwood et al., 2010). These findings suggest a central tendency of the visual system to search for greater consistency under visual constraints, such as those imposed by peripheral vision (Balas et al., 2009; Greenwood et al., 2009; Dakin et al., 2010). In this fashion, instead of combining imprecise information to form an inadequate visual representation, the visual system converges toward a more coherent representation by subtracting uneven information. In line with this multilevel account, previous studies have found distractor-related effects for both elementary features and whole object representations (Whitney and Levi, 2011). These accounts deem unlikely that this bottleneck acts upon a single and unique stage of visual processing (Levi, 2008).

#### Bistable figures

Bistable figures – e.g., Necker Cube and duck–rabbit figure – are ambiguous images that induce involuntary fluctuations between mutually exclusive interpretations. For example, staring at the Necker Cube leads to sequential changes between two perceptual views – i.e., the frontal face either oriented downward or upward (see **Figure 2**). Bistable representations reflect the inherent ambiguity conveyed by these images as our brain processes resolve these competing interpretations (Leopold and Logothetis, 1999; Kornmeier and Bach, 2012; Ishizu, 2013). Similar to interocular suppression, these figures elicit changes in visual awareness while keeping the sensory input constant. Moreover, because bistable interpretations are mutually exclusive, the perceptual dominance of one interpretation over the other leads to the complete suppression of the other one, giving researchers effective means to investigate subliminal perception. Despite its effectiveness in eliminating conscious perception, an overarching shortcoming permeates this approach: the perceptual switches triggered by ambiguous figures are scantily under the complete voluntary control of participants, reducing experimental control (Kornmeier and Bach, 2006).

It remains uncertain whether perceptual switches hinge on bottom-up or top-down mechanisms (Rach and Huster, 2014). Recognizing evidence favoring both views, hybrid accounts attempt to bridge effects related to bottom-up sensory processing, such as adaptation and fatigue, with top-down higher order processing, like anticipatory and learning factors (Long and Toppino, 2004; Toppino and Long, 2005). Specifically, the relative inability for individuals to exert total control over perceptual switches reflects bottom-up processing, whereas the capacity for observers to intentionally influence these switches demonstrates the effect of top-down processing. This view therefore emphasizes that perceptual switches stem from multilevel interactions between both lower sensory (e.g., Long et al., 1992) and higher cognitive processing (Raz et al., 2007; Knapen et al., 2011; Weilnhammer et al., 2013).

#### Motion-induced blindness

In motion-induced blindness, salient visual stimuli surrounded by global moving patterns intermittently vanish from visual awareness when participants stare at one location and covertly attend to the disappearing stimuli (see **Figure 2**; Bonneh et al., 2001). Similar to interocular suppression and bistable perception, the high experimental value of this approach largely rests on its ability to fully suppress conscious perception while keeping the sensory input constant (Scholvinck and Rees, 2009). Suppression of conscious perception through such means remains largely unpredictable as multiple factors modulate the effect (e.g., Kawabe et al., 2007; Scholvinck and Rees, 2009). Evidence suggests that suppression of perception under motion-induced blindness is unlikely to result in the pinpointing of a circumscribed brain locus (Donner et al., 2013). Supporting a multilevel account, various mechanisms have been investigated – e.g., adaptation and persistent inhibition (Gorea and Caetta, 2009), motion streak suppression (Wallis and Arnold, 2009), perceptual fill-in (Hsu et al., 2006), or depth perception ordering and surface completion (Graf et al., 2002). At the neural level, corresponding fluctuation of brain activity suggests that variations in conscious perception originate from the on-going competition between the ventral and dorsal pathways, which engage in recognition and spatial processing, respectively (Donner et al., 2008). These fluctuations appear to proceed from the competition between processing of the static targets and of the moving mask.

#### The depth of subliminal processing

Subliminal perception shows that the enduring influence of suppressed stimuli spans multiple levels of processing, including the perceptual, lexical, semantic and social. Different subliminal approaches reveal that suppression hardly affects superficial level of visual processing, such as spatial frequency, motion-direction, color, and orienting (Long and Toppino, 2004; Breitmeyer and Ögmen, 2006; Breitmeyer, 2007; Whitney and Levi, 2011; Yang and Blake, 2012; Kramer et al., 2013). A more complex picture has emerged concerning deeper levels of subliminal processing (van Gaal and Lamme, 2012). Shaping our views concerning the cognitive unconscious, subliminal processing occurs both at the cortical and subcortical level (Naccache et al., 2005). However, inconsistencies across tasks uncover task-specific differences (e.g., Faivre et al., 2012, 2014; Fogelson et al., 2014; Izatt et al., 2014). Moreover, certain discrepancies within task suggest that task-related and stimuli-related factors influence the depth of subliminal processing (e.g., CFS, Costello et al., 2009; Kang et al., 2011). We should therefore avoid to immediately reconsider the notion that certain subliminal approaches do not engage unconscious semantic processing (Gayet et al., 2014). Yet, various findings indicate that the brain subliminally processes semantic information (Costello et al., 2009; Van den Bussche et al., 2009a; Yeh et al., 2012; Sanguinetti et al., 2013). Likewise, evidence also indicates subliminal processing of faces and affective facial expressions (Jiang et al., 2007; Henson et al., 2008; Kouider et al., 2009; Sterzer et al., 2009a; Adams et al., 2010; Faivre et al., 2012; Doi and Shinohara, 2013; Izatt et al., 2014). Overall, suppression techniques have propelled a research trajectory that encompasses a large body of results. These findings indicate that unconscious processing cuts across multiple cognitive systems, emphasizing the critical role of unconscious processing. Therefore, the variety of suppression techniques often proves useful despite certain limitations.

### PRECONSCIOUS SUPPRESSION TECHNIQUES – FAILURES OF ATTENTION

#### Inattentional blindness and change blindness

Unattended, salient but unexpected events may go unnoticed (Simons and Chabris, 1999; Simons, 2000). Coined *inattentional blindness* (IB), these failures to detect prominent task-irrelevant stimuli occur when individuals engage in a demanding cognitive task (Mack and Rock, 1998). Similarly, inattentive observers can stay unaware of important changes in visual scenes, a phenomenon called *change blindness* (CB; Simons and Levin, 1997). The effects of IB primarily stem from orienting attention toward task-relevant events, preventing perceptual awareness of unattended events (Simons, 2000). Previous studies outline that several factors mediate the effects of IB, including the visual saliency and spatial location of ignored events (e.g., Koivisto et al., 2004), expectations and attentional set of the observer (e.g., Most, 2013), the difficulty of the primary-task and individual expertise (Memmert, 2006; Cartwright-Finch and Lavie, 2007), as well as inhibitory mechanisms near the fringe of the *attentional spotlight* (Thakral and Slotnick, 2010). CB, on the other hand, largely rests on interactions between attention, perception and visual short-term memory (Simons and Rensink, 2005).

Inattentional blindness (IB) and CB mainly reflect lapses of attention, wherein unattended signals lack the necessary energy and sustainability to reach conscious perception (Dehaene et al., 2006; Dehaene, 2011; Dehaene and Changeux, 2011). Both experimental techniques therefore rely on attentional failures instead of suppressive means (Kanai et al., 2010). Supporting this account, neurophysiological studies report that change detection correlates with modulation of the N2pc, an electrophysiological marker of selective attention (Eimer, 1996; Robitaille and Jolicoeur, 2006; Kiss et al., 2008; Mazza et al., 2009; Woodman et al., 2009); whereas the absence of modulation of the N2pc relates to CB (Eimer and Mazza, 2005; Busch et al., 2009; however, see Schankin and Wascher, 2007). Together, these results imply that the top-down amplification processes of selective attention prompt conscious perception of changes in the display. Conversely, in the absence of these amplification processes, sensory inputs of changes remain largely unconscious. In line with these reports, brain imaging studies of CB reveal decreased frontoparietal activity (Beck et al., 2001), a cortical network often linked with attentional processing (Corbetta et al., 2008). In addition, temporary disruption of the right parietal cortex with repetitive transcranial magnetic stimulation (rTMS) significantly impairs change detection abilities and increases CB (Beck et al., 2006; Tseng et al., 2010). Alongside attentional processing, the parietal region also associates with visual short-term memory (Berryhill and Olson, 2008). While the relationship between attention and conscious perception remains difficult to construe (van Boxtel et al., 2010; Tallon-Baudry, 2011; Chica et al., 2013), empirical findings with IB and CB techniques strongly hint that top-down amplification processes play a central role in becoming aware of sensory events.

Unattended events during IB and CB induce preconscious processing, yielding priming effects (e.g., Silverman and Mack, 2006), implicit processing of spatial information (Lathrop et al., 2011) and aversive stimuli (Wiemer et al., 2013), or tacitly influencing decision processes (Laloyaux et al., 2008). Markedly, unattended events during IB and CB induce frontal activity, suggesting deep processing despite inattention (Pessoa and Ungerleider, 2004; Thakral, 2011). However, neurophysiological results of preconscious processing remain ambiguous: whereas some studies report a fronto-central positive deflection indexing preconscious processing of unattended events (Fernandez-Duque et al., 2003; Kimura et al., 2008), results from other studies hardly show any electrophysiological component specific to preconscious processing during CB (Fernandez-Duque et al., 2003; Eimer and Mazza, 2005; Henderson and Orbach, 2006; Pourtois et al., 2006). Several task-related shortcomings limit the application of IB and CB (Kim and Blake, 2005). Importantly, once a participant learns or suspect that the display may contain otherwise covert task-irrelevant stimuli, it largely reduces the likelihood of IB and CB (Jensen et al., 2011). This issue proves particularly challenging for IB when researchers probe participants about the detection of covert events, immediately hinting the presence of concealed elements and invalidating repeated testing (Kim and Blake, 2005). This concern reduces the overall number of trials available. However, despite this liability, both IB and CB apply to a vast range of stimuli. Furthermore, these techniques possess great ecological validity, as failure to attend and detect conspicuous events reproduces outside the laboratory (e.g., Simons and Levin, 1998).

#### Attentional blink

In a stream of rapidly presented visual stimuli, attending to a task-related stimulus impairs attentional processing of subsequent stimuli at short latencies (Raymond et al., 1992). This attentional blink (AB) leads to a marked decrease in performance that underscores the limit of attentional processing and often leaves participants unaware of unattended stimuli (Shapiro et al., 1997b). Converging evidence suggest that AB largely reflects limitations of attentional capacity (for review, see Martens and Wyble, 2010). Deployed attentional resources toward the primary target temporally impede ensuing attentional processing of incoming sensory signal (Dux and Marois, 2009). Supporting this view, evidence show that greater resources devoted toward processing of the first target increase the magnitude of the AB (Arnell et al., 2007). Contrary to IB and CB, expectations hardly modulate AB, making it a highly reliable experimental design (Kim and Blake, 2005). Deep processing of non-reported targets accompanies AB. For example, unattended words facilitate ensuing processing of semantically related words (Shapiro et al., 1997a; Martens et al., 2002). Neurophysiological results also indicate that non-reported items yield modulations of the N400, an electrophysiological component indexing semantic processing (Luck et al., 1996; Rolke et al., 2001; however, see Batterink et al., 2010). However, evidence suggests that enduring preconscious processing of semantics during AB remains contingent to task demands (Giesbrecht et al., 2007). Neuroimaging results of AB indicate that unattended stimuli activate occipitotemporal regions in the near-absence of frontal activity (Marois et al., 2004; Kranczioch et al., 2005; Marti et al., 2012). In addition, brain injury to the parietal region increases the AB (Husain et al., 1997; Shapiro et al., 2002). Despite the robustness of the AB effects, this methodological paradigm relies on variation of stimuli and temporal constraints. Moreover, since these effects occur within a narrow and precise time window, researchers can hardly test them outside the laboratory. Overall, the AB represents a reliable task to investigate the underlying top-down mechanisms gating access to conscious perception in a tightly controlled fashion (e.g., Sergent et al., 2005).

### HYPNOSIS AS AN ADJUNCT TO SUBLIMINAL AND PRECONSCIOUS APPROACHES

Subliminal approaches exploit the limits of perception to suppress awareness of sensory events (Dehaene et al., 2006; Kanai et al., 2010; Dehaene and Changeux, 2011). These techniques mainly utilize competition between perceptual processing of sensory signals or representations to induce unawareness, wherein the dominance of a sensory signal or a representation prompts the suppression of subdominant ones (Blake and Logothetis, 2002). Importantly, while attentional processing moderates subliminal processing (Naccache et al., 2002; Kiefer and Brendel, 2006; Kiefer and Martens, 2010; Martens et al., 2011), conscious suppression hardly involves top-down factors. Instead, the effects of subliminal processing stem from weakened sensory signals and subdominant perceptual representations. Accordingly, subliminal approaches hinge on perceptual failures. Conversely, preconscious approaches rests on the disruption of top-down amplification processes, thereby preventing conscious access to sensory events (Naccache et al., 2002; Dehaene et al., 2006; Kanai et al., 2010). As a result, this approach may involve the processing of sensory signals strong enough for global broadcast, yet these signals remain incapable of surpassing the threshold of consciousness without the necessary top-down amplification (Dehaene et al., 2006). In this way, preconscious processing reflects attentional failures. The distinction between subliminal and preconscious approaches represents a reliable taxonomy of unconscious processing based on the differences between perceptual and attentional failures (**Figure 1**; Kanai et al., 2010).

The broad range of mechanisms selectively engaged by each of the abovementioned methods challenges our capacity to generalize findings across different tasks. As we explained, these techniques yield important findings about the scope and depth of subliminal and preconscious processing. Notably, bottom-up approaches afford researchers with plentiful experimental control, yet offer limited ecological validity. Conversely, top-down approaches, such as IB and CB, propose an ecological tactic to investigate unconscious processing (Simons and Levin, 1997; Simons, 2000; Simons and Rensink, 2005; Jensen et al., 2011), but remain experimentally challenged by top-down factors. For example, the popular invisible gorilla paradigm represents a compelling framework that generalizes to everyday tasks (Simons and Chabris, 1999), yet suffers from limited empirical control and methodological practicality (Kim and Blake, 2005). These key observations shape the trajectory of current research on subliminal and preconscious research. Moreover, they raise important empirical and theoretical questions about our ability to bridge the gap between these different methods. Here we submit that instrumental hypnosis – a top-down approach, which relies on higher cognitive functions regulating the downstream operations of the perceptual and affective systems – offers new investigative prospects to elucidate the unconscious mind. Moreover we argue that this unique approach transcends the subliminal versus preconscious taxonomy, as hypnosis can induce perceptual and attentional failures. Overall, hypnosis provides the means to replicate established findings and explore new hypotheses.

**FIGURE 3:**
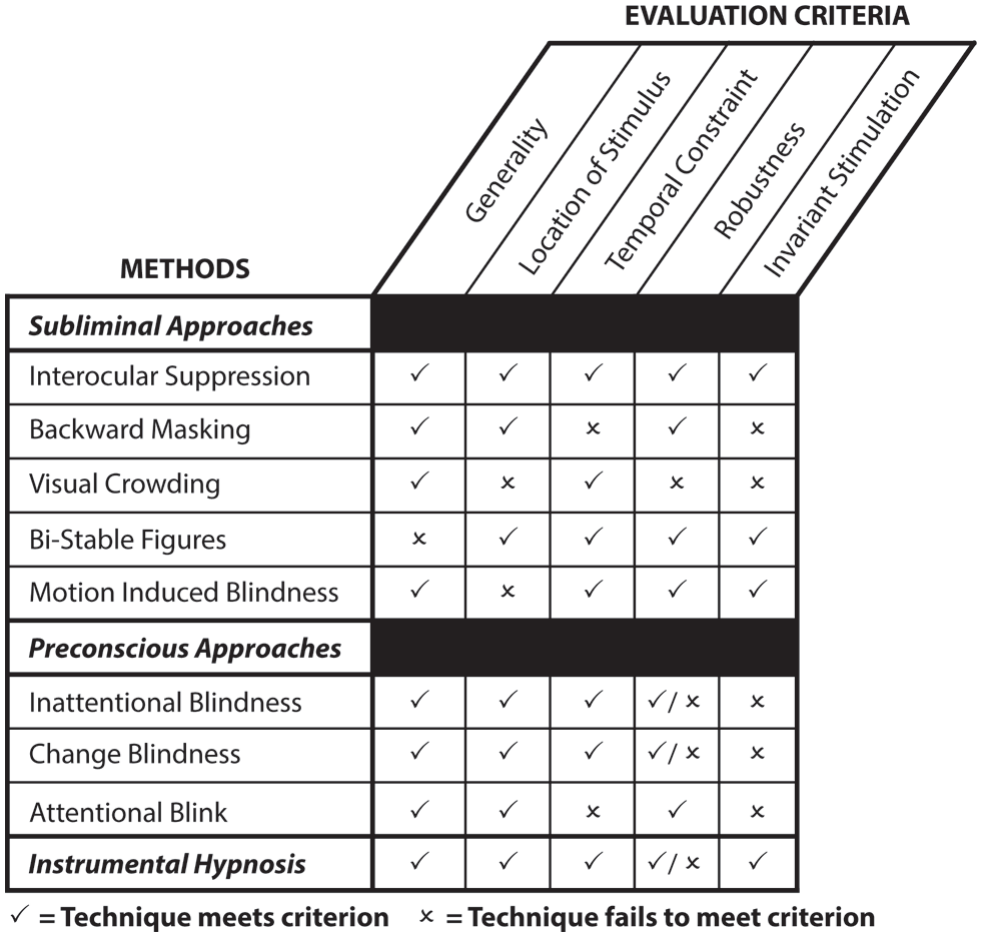
**Strengths and weaknesses of contemporary techniques to investigate unconscious perception and instrumental hypnosis as a function of evaluation criteria.** *Generality*: whether the technique applies to a broad range of stimuli or only to a selected few. *Location of stimulus*: whether the stimulus has to be presented at the center or the periphery of the visual field to induce conscious suppression or inattention. *Temporal constraint*: whether the technique imposes a temporal constraint relative to the duration of the stimulus presentation. *Robustness*: whether the technique completely abolishes awareness. *Invariant stimulation*: whether conscious suppression requires significant modifications of sensory events to make a stimulus invisible. A “✓” indicates that the technique meets this particular criterion, whereas an “×” indicates that the technique fails to meet this particular criterion.

To assess the aforementioned techniques (see **Figure 3**), we follow the criteria put forth in the literature (Kim and Blake, 2005). This set of criteria evaluates the efficacy of each technique and gauges the potential of experimental methods to generate reliable and valid findings concerning unconscious processes:

(i) *Generality*: whether the technique applies to a broad range of stimuli or only to a select few.
(ii) *Stimulus location*: whether the stimulus has to be presented at the center or the periphery of the visual field.
(iii) *Temporal constraint*: whether the technique imposes a temporal constraint relative to the duration of the stimulus presentation.
(iv) *Robustness*: whether this technique completely abolishes awareness.
(v) *Invariant stimulation*: whether conscious suppression requires significant modifications of sensory events to make a stimulus invisible – e.g., adding a mask to induce conscious suppression during backward masking.

## PART III – USING HYPNOSIS TO INVESTIGATE THE UNCONSCIOUS MIND

### HYPNOSIS: A TOP-DOWN APPROACH TO INVESTIGATE THE UNCONSCIOUS MIND

Hypnosis represents an increasingly popular area of research in cognitive science, including notable ventures in the domains of perception, attention, memory, and motor control (Nash and Barnier, 2008). For example, hypnotic suggestions represent central vehicles in exploring the notion of automatic processing and induce de-automatization of ballistic responses in the Stroop, McGurk, and Simon effects (Raz et al., 2002, 2005; Iani et al., 2006; Lifshitz et al., 2013; Déry et al., 2014). Within this growing field of research, scholars and clinicians conceptualize the scientific investigation of hypnosis in a dichotomous fashion, differentiating intrinsic research on hypnosis, which focuses on the phenomenon itself, from an instrumental approach, where researchers employ hypnosis as an experimental tool to investigate cognition (Oakley and Halligan, 2009, 2013). Our view focuses on supplementing current experimental methodologies with this instrumental strategy to further unravel the cognitive unconscious.

Theoretical frameworks for hypnosis largely cluster around the appellations of state and non-state models. State theories posit that hypnosis implies a particular psychological state – e.g., an altered state of consciousness – whereas non-state theories typically argue that hypnosis essentially reduces to sociocognitive factors such as motivation and compliance (Kirsch and Lynn, 1995; Kallio and Revonsuo, 2003; Kirsch, 2011; Raz, 2011a; Mazzoni et al., 2013). In spite of this conceptual distinction, the use of hypnosis often includes an induction phase to increase mental absorption followed by a suggestion phase providing directions to elicit particular changes in thoughts and behaviors. Hypnotic responses usually result from hypnotic suggestions. The degree of responsiveness to hypnotic suggestions represents a robust measure with normal distribution and high test-retest reliability (Piccione et al., 1989). Highly hypnotically suggestible individuals (HHSs), as opposed to low hypnotically suggestible individuals (LHSs), characteristically respond to “cognitive” suggestions – i.e., suggestions that involve changes in perception and memory (Kirsch et al., 1999). Accordingly, researchers often compare the performances of HHSs and LHSs to demonstrate the effects of hypnosis (Nash and Barnier, 2008).

Top-down regulatory processes – e.g., attention, cognitive control and monitoring – play a central role in mediating responses to hypnotic suggestions (Crawford, 1994; Gruzelier, 1998; Raz, 2004, 2011b; Egner and Raz, 2007; Dienes, 2012; Lifshitz et al., 2012; Dienes and Hutton, 2013). Specifically, hypnosis modulates top-down processes to dramatically change the implementation of cognitive strategies during hypnotic responses (Egner and Raz, 2007). Furthermore, the execution of hypnotic responses often appears dissociated from voluntary control, as they generally feel involuntary and effortless (Spanos et al., 1977). This phenomenological aspect represents a critical component of hypnotic phenomena (Kirsch and Lynn, 1998). A family of prevalent theories contends that this central property of hypnosis mainly reflects decoupling between cognitive control and monitoring processes (cf., Jamieson and Woody, 2007; Woody and Sadler, 2008). According to this view, hypnosis not only alters cognitive control but also modifies the supervision of these control processes. Supporting this view, a neuroimaging study of HHSs report a functional disconnection between the lateral prefrontal cortex, often linked to cognitive control processes, and the anterior cingulate cortex (ACC), a brain region associated with cognitive monitoring (Egner et al., 2005). This finding echoes numerous brain imagining studies of hypnosis that show similar modulations of the ACC in the absence of specific hypnotic suggestion (Faymonville et al., 2000, 2003; Rainville et al., 2002; McGeown et al., 2009; Vanhaudenhuyse et al., 2009; Deeley et al., 2012; Müller et al., 2012, 2013).

Emphasizing the importance of individual variability, compliant participants frequently report using different cognitive strategies to successfully respond to the very same suggestion (McConkey et al., 1989; Heap et al., 2004). This inter-individual variability in hypnotic responses raises questions concerning the link between specific cognitive styles and hypnotic susceptibility, which hints that specific sub-types of cognitive profiles could enable greater hypnotic responses (Terhune et al., 2011; Brown and Oakley, 2004). In this respect, some scholars argue that what characterizes HHSs is their greater cognitive flexibility (Crawford, 1994; Gruzelier, 1998); others regard the improvement in attention and inhibitory control as a near-universal outcome (Dienes et al., 2009; Varga et al., 2011). Supporting the cognitive flexibility view, neuroimaging findings from HHSs show increased functional connectivity between the dorsolateral prefrontal cortex (DLPFC), a cortical region strongly associated with cognitive control, and saliency networks, which likely mediate somatic, automatic, and emotional information (Hoeft et al., 2012). However, a recent study report that temporary disruption of the DLPFC with rTMS also causes modifications of hypnotic responses, hinting that hypnosis could reflect the disruption of cognitive control and monitoring (Dienes and Hutton, 2013). Resting-state brain imaging studies show that HHSs show decreased activity in the anterior part of default mode network (DMN), a brain network negatively correlated with goal-directed activity (McGeown et al., 2009). Reduced activity in the anterior part of DMN may therefore indicate a propensity to engage in goal-driven behaviors – i.e., the mental preparation to comply with hypnotic suggestions and produce hypnotic responses. Other studies also report a significant change in DMN activity related to hypnosis (Demertzi et al., 2011; Deeley et al., 2012; Lipari et al., 2012). Taken together, these cumulative findings intimate the importance of top-down regulatory functions in hypnotic phenomena.

### HYPNOSIS AS A VEHICLE TO UNCOVER THE UNCONSCIOUS MIND

**FIGURE 4:**
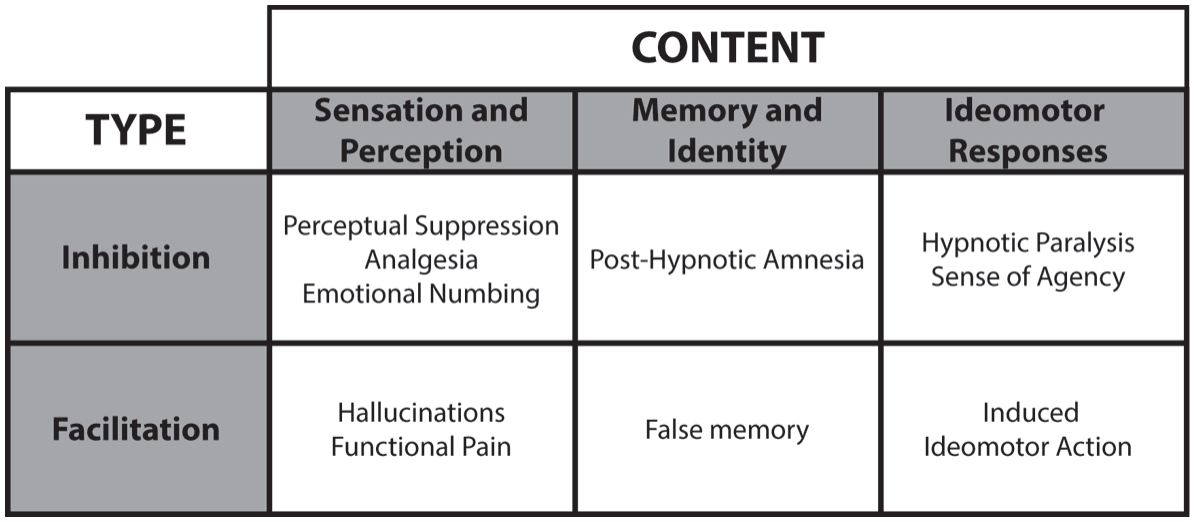
**Hypnotic suggestions divide as a function of type and content.** These various hypnotic suggestions yield numerous hypnotic effects.

**FIGURE 5:**
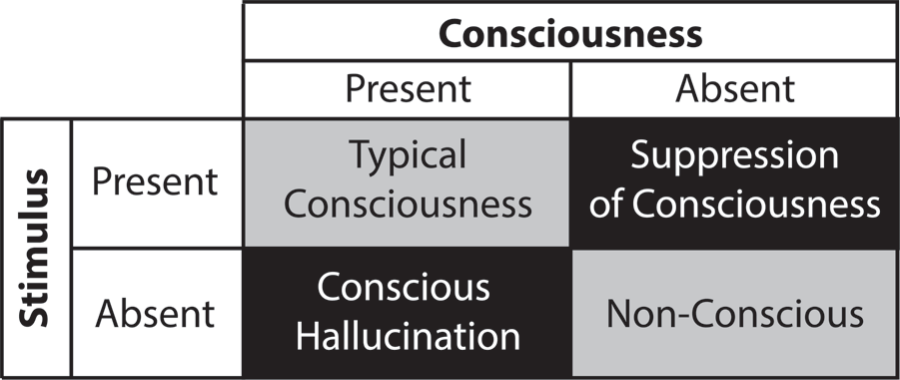
**Balanced experimental design using instrumental hypnosis where contrast between conscious suppression – i.e., stimulus is present and consciousness is absent – and conscious hallucination – i.e., stimulus is absent and consciousness is present – enable targeting of neural correlates of consciousness**.

Hypnotic suggestions divide as a function of type and content (see **Figure 4**; Woody and Sadler, 2008). Within this framework, suggestions either facilitate or suppress cognitions and behaviors. For example, facilitation may yield hallucinations (e.g., Bryant and Mallard, 2003), whereas suppression can interfere with consciousness (e.g., Bryant and Kourch, 2001). Accordingly, researchers can test conscious and unconscious processing in a fully orthogonal manner (see **Figure 5**), a significant experimental benefit to better isolate the NCC. The content of hypnotic suggestions selectively targets specific mental functions and behaviors. Thus, we will demonstrate how hypnosis encompasses a wide variety of experimental possibilities to investigate unconscious processes (Oakley and Halligan, 2009, 2013; Cox and Barnier, 2010; Bortolotti et al., 2012). Importantly, because hypnotic suggestions can either induce suppression of consciousness or influence attentional processing to impede top-down amplification, this top-down approach bridge the subliminal versus preconscious dichotomy (see **Figure 1**). Here we discuss several avenues based on such research developments.

#### Sensation and perception

Hypnosis selectively targets and modifies perception of sensory events. For example, it can alter perception of colors (Kosslyn et al., 2000; Mallard and Bryant, 2001; Spiegel, 2003; McGeown et al., 2009; Kallio and Koivisto, 2013; Koivisto et al., 2013), induce compelling experiences of grapheme-color synesthesia – a condition characterized by perceptual experiences of anomalous combinations of cross-modal sensations (Cohen Kadosh et al., 2009; however, see Anderson et al., 2014), and even temporarily abolish co-occurrences of secondary sensory experiences in synesthetes (Terhune et al., 2010).^1^ Indicating the reliability and sustainability of these remarkable changes, hypnotically induced alteration of color perception correlates with corresponding modifications in neural response (Kosslyn et al., 2000; McGeown et al., 2012). Specifically, neuroimaging results of hypnotically induced altered perception show modulation of brain regions associated with color processing – i.e., bilateral fusiform gyrus, primary visual area (Kosslyn et al., 2000; McGeown et al., 2012). Importantly, this phenomenon raises intriguing questions regarding the study of subliminal processing, because such changes in perception entail the suppression from consciousness of the actual sensory event. For example, would suppressing green sensory input by supplanting it with hypnotically induced hallucinations produce green-related priming effects? The affirmative would support the subliminal processing of hypnotically induced suppressed content. Such questions bear on the investigation of top-down-induced subliminal processing. Recent neurophysiological investigations found that alteration of color perception correlates with modulations of neural oscillatory activity over posterior regions (Koivisto et al., 2013). These modulations of neuronal responses submit the idea of an early mechanism involved in probing the stored mental representation of the suggested color and in the modification of the ensuing percept. Consistent with the idea that hypnotic suggestions to alter color perception operate preconsciously, participants barely detect perceptual or sensory changes, suggesting that these alterations precede conscious access (Kallio and Koivisto, 2013). Suppression of sensory signals arguably precedes the global broadcast. Supporting this early top-down effect on sensory input, event-related potentials indicate hypnotic modulations of primary visual components (i.e., P1 and N1; Raz et al., 2005).

Hypnosis also modulates phenomenological aspects of conscious experience, such as pain perception (Patterson and Jensen, 2003; Jensen and Patterson, 2006; Price and Rainville, 2013). Called hypnotic analgesia, this phenomenon does not follow from the release of endogenous analgesics (Goldstein and Hilgard, 1975) or an increased state of relaxation (Miller et al., 1991). Instead, hypnotic analgesia arguably originates from various factors, including the alteration of expectations relative to impending painful events, as well as attentional and emotional regulation mechanisms (Kiernan et al., 1995; Rainville et al., 1999a; Ploghaus et al., 2003; Koyama et al., 2005; Price and Rainville, 2013). Hypnotic analgesia triggers pain-related inhibitory neural mechanisms (Vanhaudenhuyse et al., 2009). Similar to color perception, these changes in perception demonstrate how hypnosis elicits powerful effects over perceptual experience. Brain imaging studies of this phenomenon underline a dissociation between the somatosensory cortex, involved in processing of nociceptive signals, and the ACC, a region associated with conscious access to pain sensation (Rainville et al., 1997, 1999b, 2002; Faymonville et al., 2000, 2003; Hofbauer et al., 2001). Grounded in this functional dissociation between sensory and affective components of pain (Rainville et al., 1999a), current findings suggest that alteration of pain perception can either proceed from direct interferences of sensory processing (Hofbauer et al., 2001), akin to subliminal approaches, or by modulating conscious access to pain sensation (Rainville et al., 2002), comparable to preconscious approaches. In line with this view, neurophysiological results imply that hypnotic analgesia affects early as well as late stages of nociceptive processing (De Pascalis et al., 2008). Analogous to the color-hallucination paradigm, such changes in pain perception raise important questions concerning the effects of unconscious nociceptive processing on behavior. For example, would unconscious processing of nociceptive stimuli still instigate a level of discomfort? Moreover, in addition to analgesia, hypnotic suggestions can also trigger functional pain – i.e., the subjective experience of pain in the absence of a noxious stimulus (Derbyshire et al., 2004). This functional aspect of hypnosis brings about the experimental ability to compare, in a balanced design, conscious perception in the absence of a stimulus and the lack of conscious perception in the presence of a stimulus, in order to effectively isolate the NCC (see **Figure 5**).

#### Memory and identity

Posthypnotic amnesia (PHA) represents memory lapses of events that took place under hypnosis, after termination of hypnotic induction (Kihlstrom, 1985, 1997; Barnier, 2002a). Affording researchers with increased experimental control, these memory deficits contributed to the development of experimental research on implicit cognition (Barnier et al., 2001). Importantly, prearranged post-hypnotic cues induce recall, implying that memory lapses mainly reflect the inability to access and retrieve stored information rather than encoding and storage deficits (Geiselman et al., 1983; Kihlstrom, 1997). Hence, PHA putatively originates from top-down failures to access and retrieve information, relating this phenomenon to preconscious approaches. The underlying neurophysiological correlates of PHA involve the modulations of attentional processes relative to access and selection of stored information (Allen et al., 1995; Schnyer and Allen, 1995). In addition, compared to normal retrieval of stored information, PHA correlates with decreased activity in the extrastriate and temporal cortical regions, as well as increased activity in the rostral lateral PFC (Mendelsohn et al., 2008). This reduced activity in the temporal lobes likely reflects the incapacity to successful access stored information, as this brain region strongly associates with long-term memory storage (Wixted and Squire, 2011). Hypothetically, increased PFC activity could reflect the implementation of hypnotic responses to actively hinder retrieval processes.

Past research shows that temporarily irretrievable material influences behavior nonetheless (Kihlstrom, 1980; Spanos et al., 1982; Kinnunen and Zamansky, 1996; Bryant et al., 1999; Barnier et al., 2001). For example, reflecting the distinction between explicit and implicit memory systems, performances of HHSs on a word association task denote PHA-related priming effects despite significant deficits on explicit recall (Kihlstrom, 1980; David et al., 2000; Barnier et al., 2001). PHA experiments also reveal suppression of conscious access to episodic memory (Kihlstrom, 1997), source memory (Evans and Kihlstrom, 1973; Evans, 1979), and even autobiographical memory (Barnier and McConkey, 1999; Barnier, 2002a,b; Cox and Barnier, 2003; Barnier et al., 2004). Notably, suppression of access to autobiographical memories may lead to significant effects on personal identity (Barnier, 2002b). These examples illustrate how PHA offers a unique framework to test various hypotheses on the cognitive unconscious beyond perceptual processing.

Contrary to PHA, few studies looked at the effects of hypnotic agnosia – i.e., the functional inability to access semantic knowledge (Kihlstrom, 1997; Raz, 2011b). This research gap leaves open numerous experimental possibilities to probe unconscious semantic processing using hypnosis, stretching from the semantic categories of inaccessible items to modality specific deficits. Furthermore, the case of hypnotic agnosia evokes an intriguing paradox wherein the selective interference to access a particular semantic content requires the ability to minimally identify that content at some level – e.g., the hypnotically induced discriminating inability to recognize scissors, requires the tacit ability to discriminate scissors from other objects. This phenomenon therefore demonstrates how top-down processing may act through tacit knowledge – i.e., knowledge in the absence of awareness.

#### Ideomotor response

Hypnosis can decouple volitions and actions (Halligan et al., 2000; Blakemore et al., 2003; Ward et al., 2003; Cojan et al., 2009; Cardeña et al., 2012; Coutlee and Huettel, 2012; Peter et al., 2012; Deeley et al., 2013a,b, 2014; however, see Haggard et al., 2004). Hypnotic suggestions directly targeted at the sense of control disrupt willed actions and induce alien control. For example, during involuntary arm levitation, responsive participants raise their arm in the absence of conscious control (Blakemore et al., 2003). This hypnotic effect reduces overall muscle activity (Peter et al., 2012) and relates to significant changes in the cerebellar-parietal network (Blakemore et al., 2003). These results parallel brain-imaging studies that report modulation of parietal activation during hypnotically induced paralysis, wherein participants experience the inability to move a limb (Cojan et al., 2009; Cardeña et al., 2012; Coutlee and Huettel, 2012; Deeley et al., 2013a). Investigating the effects of hypnotic suggestion on the perception of voluntary and involuntary movements, a recent neuroimaging study reports that loss of perceived control correlates with decreased connectivity between the supplementary motor area, associated with motor planning, and the primary motor area (Deeley et al., 2013a). These results suggest that decoupling the planning and the implementation of actions decreases the feeling of control during movements. Additional results from this study also indicate that reduced conscious perception of involuntary actions correlates with decrease neural activity of the parietal lobe, suggesting that modulation of parietal activity relates more strongly with awareness of movements than feeling of control. In a separate study, the same research group investigated involuntary movements as a function of locus of control (Deeley et al., 2014). Results show that induced involuntary control may reflect various types of alien control and modulations of agency. Thus, various strategies may interfere with conscious access to feelings of control. Together, these findings highlight how ideomotor suggestions elicit important interactions between hypnotic response, awareness of movement and locus of control. Moreover, they also show how conscious access to the control of movements influences the phenomenology of action.

#### Thought suppression and hypnotically induced clinical analogs

Intrusive cognitions and emotions often accompany psychopathology (Wenzlaff and Wegner, 2000). In order to aid patients, clinicians use hypnosis to suppress unwanted thoughts (Bryant and Wimalaweera, 2006; Bryant and Sindicich, 2007). Moreover, hypnotic suggestions can also numb the conscious perception of unpleasant emotions (Bryant and Kourch, 2001; Bryant and Mallard, 2002; Bryant, 2005; Bryant and Kapur, 2006; Bryant and Fearns, 2007; Sebastiani et al., 2007). Experimental results show that hypnotic numbing of emotions significantly reduces emotional and somatic responses to aversive stimuli (Bryant and Kourch, 2001; Bryant and Mallard, 2002). Furthermore, emphasizing the accuracy of hypnotic suggestions, evidence also indicates that emotional suppression solely interferes with affective dimensions of cognition, leaving the cognitive content available for conscious processing (Bryant and Fearns, 2007). Interestingly, an experimental study investigated the interactions between masked-induced and hypnotically induced suppression mechanisms. Using a backward masking design, results show that hypnotically induced emotional numbing suppresses subliminal processing of masked aversive stimuli, thereby demonstrating that hypnotic suppression of emotions occurs at the unconscious level – i.e., prior to global broadcast (Bryant, 2005). Hence, hypnotic suppression acts early and can supersede subliminal processing. Together, hypnotic suppression of thoughts and emotions provide a reliable and distinctive framework to investigate subliminal processing.

In experimental psychopathology, hypnotic suggestions target specific functions and dramatically influence cognitions and behaviors (Oakley, 2006; Cox and Barnier, 2010; Woody and Szechtman, 2011; Bortolotti et al., 2012). For example, one study used hypnosis to interfere with subjective feelings associated with task completion and motivational security, producing obsessive-compulsive-like behaviors in typical participants (Woody et al., 2005). This study underlines the importance of conscious access to certain affective signals in the phenomenology of even the utmost mundane tasks – e.g., washing your hands. In the same vein, hypnosis can also eliminate conscious access to selfhood-related information, yielding mirrored-self misidentification delusions – a monothematic delusion characterized by the inability to recognize self-reflections in the mirror (Barnier et al., 2010; Connors et al., 2012a,b, 2013). Evidence shows that this induced delusion stems from faces recognition impairment (Connors et al., 2012a, 2013). Critically, hypnotically induced mirror agnosia – i.e., unavailability of knowledge about mirrors – also facilitates the generation of mirrored-self misidentification analogs (Connors et al., 2012b). Aside from exploring new hypotheses, research with hypnotically induced clinical analogs underlines the importance of conscious access to various sources of information, such as sense of completion or selfhood-related recognition. From this perspective, conscious suppression not only provides critical information about the unconscious mind, but also helps to identify the functional role of various processes related to consciousness by looking at hypnotically induced maladapted behaviors and delusions.

The fields of neuropsychology and behavioral neurology often feature deficits that are amenable to top-down influences (Weiskrantz, 1986; Cowey, 2010; Overgaard, 2011) at different levels (Marshall and Halligan, 1995; Fink et al., 1996). Experimental accounts of hypnosis show how hypnotic suggestions can induce reversible neuropsychological conditions – a form of behavioral analog to TMS (cf., Raz and Wolfson, 2010). One example is visuospatial hemineglect, where hypnotic suggestions to favor one visual hemifield over the other lead to significant decreases in visual performance on the neglected side and neglect-like symptoms (Oakley and Halligan, 2009, see supplementary material; Raz, 2004; Priftis et al., 2011). In accordance with neuropsychological findings that show distinctive levels of unconscious processing, e.g., evidence from visuospatial neglect reveals processing of coarse global representation in the absence awareness (Marshall and Halligan, 1995), hypnotically induced neglect can reliably expand this line of research. Similar to prevailing preconscious approaches, this research strategy underlines the experimental potential of hypnosis to foster critical information about the link between orienting of attention and visual awareness, and opens novel avenues to investigate the preconscious processing of unattended stimuli.

#### The experimental potential of hypnosis

Whether hypnosis acts through suppressive means or influences attention to impede conscious access, this top-down methodological approach possesses formidable potential to study the unconscious mind. Two general features make hypnosis a unique approach. First, hypnotic suggestions afford researchers with a wide spectrum of experimental possibilities. Second, whereas the prevailing approaches either take advantage of perceptual limitations or interfere with top-down amplification processes, hypnosis harness top-down processes to investigate both subliminal and preconscious phenomena. Indeed, due to the variety of hypnotic suggestions, hypnosis can prompt perceptual and attentional failures. Also, the accuracy of hypnosis (Raz and Michels, 2007) allows researchers to selectively target mechanisms gating access to consciousness.

As illustrated previously, hypnotic phenomena comprise numerous brain systems, depending on the content of the hypnotic suggestion and the targeted function. Therefore hypnotic suggestions act through various means: while certain suggestions engage suppression mechanisms and yield subliminal processing, other suggestions interfere with the deployment of top-down amplification and elicit preconscious processing (see **Figure 1**). During hypnotically induced subliminal and preconscious processing, hypnotic responses recruit frontal networks implicated in top-down attentional regulation, control and monitoring processes (Rainville et al., 1999b; Casale et al., 2012; Kihlstrom, 2013; Oakley and Halligan, 2013). As mentioned previously, these brain regions associate with the implementation of cognitive strategies to successfully comply with hypnotic suggestions. Subsequent neural effects putatively reflect the targeted function of the hypnotic suggestion (Oakley, 2008). For example, alterations of colour perception correspond with significant changes in the visual areas (Kosslyn et al., 2000; McGeown et al., 2012) and oscillatory modulations of posterior brain activity 70 to 120 milliseconds post stimulus onset (Koivisto et al., 2013). These results suggest the presence of an early mechanism that supplants the actual representation of sensory events with the suggestion-related stored representation, subsequently producing alteration of perception and suppressing sensory input. In addition, because hypnosis supposedly elicits modifications of monitoring processes, perceptual alterations could also involve modifications of reality monitoring – i.e., the cognitive ability to assess the authenticity of changes in perception (Bryant and Mallard, 2003, 2005). Contemporary subliminal approaches and hypnotic approach therefore encompass different suppression mechanisms. Whereas the former exploits perceptual limitations, the latter use top-down mechanisms to suppress conscious perception. Conversely, hypnotically induced preconscious processing resembles prevailing preconscious approaches. For example, hypnotic responses can also orient attention away from sensory events, thereby impeding top-down amplification of sensory signals (Raz, 2004; Oakley and Halligan, 2009; Priftis et al., 2011). In addition, heightened mental absorption during hypnosis (Rainville et al., 2002) could tax attentional resources, triggering similar effects to the AB. In summary, the hypnotic approach to elucidate unconscious processing rests on a broad variety of mechanisms. This wide spectrum offers various experimental possibilities that overlap both subliminal and preconscious processing.

Overall, the use of hypnosis to investigate the cognitive unconscious compares favorably to contemporary methodologies (see **Figure 3**): this approach applies to a broad range of visual and non-visual stimuli; works equally well for stimuli presented centrally or peripherally; hardly necessitates temporal constraint relative to the presentation of the stimulus or variation in sensory events. Finally, various experiments imply the robustness of unconscious hypnotic phenomena, even if the phenomenological dimensions of hypnosis remain roughly defined (Rainville and Price, 2003; Jamieson, 2007). This approach also offer the following advantages: first, because it yields subliminal or preconscious processing while keeping sensory inputs constant, this technique provides researchers with greater experimental validity to isolate conscious from unconscious processing. As mentioned previously, this feature invites direct comparisons between conscious processing and unconscious processing without introducing confounding variables relative to changes in the sensory input. Second, hypnosis may selectively suppress certain content from conscious experience – e.g., emotions – without altering the whole perceptual experience. This methodological benefit becomes particularly useful in the context of concurrent presentations of sensory events. In addition, hypnosis may harness the ecological benefits of preconscious approaches. Finally, this approach may also be used in conjunction with other suppression methods; a feature that expands the methodological possibilities through the various combinations it creates. Exemplifying this malleability, HHSs exhibit distinctive response patterns to masked primes (Bryant, 2005). In comparisons to other techniques, hypnosis therefore represents a valid and reliable instrument to probe the unconscious mind.

Despite these benefits, certain obstacles to the use of hypnosis in the context of the suppression of consciousness might arise. Here we address some of these concerns. First, HHSs are often carefully selected in hypnosis experiments to demonstrate the full potential of hypnotic suggestions (Hilgard, 1965), despite constituting only 10 to 15% of the population. This situation entails that interpretations of such experiments might not generalize and could merely reflect certain psychological characteristics of this particular group of individuals. A similar concern pertains to the fact that certain scholars consider hypnosis as a specific form of altered consciousness, which suggests that the effects of hypnosis might reduce to this specific altered mental state, again hindering generalizability. However, the notion that hypnosis implies a particular mental state remains highly debatable (Kirsch and Lynn, 1995; Kirsch, 2011). More importantly, both objections fail to apply to the instrumental approach, wherein hypnosis serves as an experimental tool to investigate cognition, and do not focus on hypnosis by itself. In the instrumental context, psychometric specificities of hypnosis are typically disregarded because they hardly provide insight into the model or hypothesis being tested. For example, the application of instrumental hypnosis to investigate the notion of automaticity proposes novel perspectives about this central psychological construct regardless of psychometric characteristics of hypnosis (Raz et al., 2002, 2005; Iani et al., 2006; Campbell et al., 2012; Lifshitz et al., 2013). For this reason, questions about generalizability are mostly irrelevant. A third concern follows from inter-individual variability in hypnotic responses, an epistemological obstacle that highlights the heterogeneous nature of responsiveness. Despite the importance of taking this aspect into consideration, this variability among individuals only calls for precautions when it comes to interpreting the data. In addition, qualitative data could properly assess and control for this variability. Indeed, a growing array of interviewing techniques, such as the elicitation interview, provide tools for identifying cognitive strategies (Vermersch, 1994; Le Van Quyen and Petitmengin, 2002).

A final concern pertains to the objective control of subjects’ awareness, a central issue that transcends research on conscious and unconscious processes (Seth et al., 2008; Overgaard and Timmermans, 2010). Alongside subjective reports, the subliminal and preconscious approaches typically control for conscious perception by ensuring that unconscious-related performances remain at chance level (Kouider and Dehaene, 2007). These performance-based strategies, however, often miscalculate conscious perception because subjective reports may vary while objective measures stay constant (Lau and Passingham, 2006). Optimally, research involving hypnosis requires two fundamental contrasts: hypnotic versus non-hypnotic experimental conditions, as well as HHSs versus LHSs. These comparisons provide the means to properly screen for, measure the effects of, and thereby bolster the effects of hypnotic suggestions (Mazzoni et al., 2013). Subsequently, two pivotal strategies likely enable better control of awareness. First, researchers may use concomitant objective measures to the primary task. For example, during emotional numbing, somatic measures corroborate emotional suppression (Bryant and Mallard, 2002). However, this strategy largely assumes that concomitant objective measures represent a tight control for subjective experience – an unwarranted assumption. Because they rarely represent an infallible control of awareness (Sandberg et al., 2010), concomitant objective measures only propose convergent evidence. Second, researchers may control for hypnotic effects using a secondary task; for example, Stroop (MacLeod, 1991) or color-based digit detection (Cohen Kadosh et al., 2009) may control for alterations of color perception. In the absence of robust control strategies, converging evidence from multiple measures represents the best strategy to remedy this lacuna (Seth et al., 2008).

## CONCLUSION

Here we herald instrumental hypnosis as a new experimental vehicle to probe the structure and functioning of the cognitive unconscious. Whereas most current techniques investigate the unconscious mind via subliminal approaches that challenge our perceptual limitations and preconscious approaches that rest on inattention, the hypnosis lens facilitates both suppression and inattention via top-down mechanisms. Beyond the empirical potential to explore novel ideas and hypotheses, top-down control provides scientists with increased experimental flexibility by allowing target processing of specific sensory events. Moreover, hypnotic hallucinations provide an efficient means to capture the NCC using a full two-by-two balanced design allowing for a direct comparison of conscious and unconscious conditions. Thus, scholars stand to benefit from the use of hypnosis in their quest to better understand the underpinnings of the unconscious mind (Raz, 2011b). Incorporating this tool into the armamentarium available to investigators of the cognitive unconscious will likely pave the road to a more encompassing scientific understanding of this budding field.

## Conflict of Interest Statement

The authors declare that the research was conducted in the absence of any commercial or financial relationships that could be construed as a potential conflict of interest.

## Footnotes

1 Hypnotically induced blindness could represent another case of conscious suppression, however the phenomenological status of these phenomena remains rather ambiguous (Oakley and Halligan, 2009).
